# Is one enough? The case for non-additive influences of visual features on crossmodal Stroop interference

**DOI:** 10.3389/fpsyg.2013.00799

**Published:** 2013-10-31

**Authors:** Lawrence G. Appelbaum, Sarah E. Donohue, Christina J. Park, Marty G. Woldorff

**Affiliations:** ^1^Department of Psychiatry and Behavioral Sciences, Duke UniversityDurham, NC, USA; ^2^Center for Cognitive Neuroscience, Duke UniversityDurham, NC, USA; ^3^Department of Neurobiology, Duke UniversityDurham, NC, USA; ^4^Department of Psychology and Neuroscience, Duke UniversityDurham, NC, USA

**Keywords:** multisensory conflict, stroop task, redundancy gains, stimulus onset asynchrony (SOA)

## Abstract

When different perceptual signals arising from the same physical entity are integrated, they form a more reliable sensory estimate. When such repetitive sensory signals are pitted against other competing stimuli, such as in a Stroop Task, this redundancy may lead to stronger processing that biases behavior toward reporting the redundant stimuli. This bias would therefore, be expected to evoke greater incongruency effects than if these stimuli did not contain redundant sensory features. In the present paper we report that this is not the case for a set of three crossmodal, auditory-visual Stroop tasks. In these tasks participants attended to, and reported, either the visual or the auditory stimulus (in separate blocks) while ignoring the other, unattended modality. The visual component of these stimuli could be purely semantic (words), purely perceptual (colors), or the combination of both. Based on previous work showing enhanced crossmodal integration and visual search gains for redundantly coded stimuli, we had expected that relative to the single features, redundant visual features would have induced both greater visual distracter incongruency effects for attended auditory targets, and been less influenced by auditory distracters for attended visual targets. Overall, reaction times were faster for visual targets and were dominated by behavioral facilitation for the cross-modal interactions (relative to interference), but showed surprisingly little influence of visual feature redundancy. Post-hoc analyses revealed modest and trending evidence for possible increases in behavioral interference for redundant visual distracters on auditory targets, however, these effects were substantially smaller than anticipated and were not accompanied by a redundancy effect for behavioral facilitation or for attended visual targets.

## Introduction

Sensory signals are inherently noisy and therefore, redundant information is highly useful in reducing low-level variance present in the sensory signal and improving perception. For example, it is easier to judge the shape of an object that can be seen and felt, relative to making judgments based on either sense alone. Numerous empirical studies have shown that when different perceptual signals of the same physical entity are integrated, they form a more reliable sensory percept (Landy and Kojima, 2001; Ernst and Banks, 2002; Knill and Saunders, 2003; Roach et al., 2006; Beierholm et al., 2009).

Computational models of information processing propose that multisensory integration produces a weighted average of various sensory signals that are available. Under such maximum likelihood estimation (MLE) frameworks, the contribution of each sensory input to the ultimate percept is determined by the relative reliability of the information it provides (van Beers et al., 1999; Ernst and Banks, 2002; Hillis et al., 2002; Gepshtein and Banks, 2003; Alais and Burr, 2004; Roach et al., 2006; Beierholm et al., 2009). By accounting for prior experience with certain types of sensory inputs, such MLE approaches have been able to explain a number of classic demonstrations in which multisensory judgments are biased in favor of more reliable sensory signals. For example, based on prior information about the environment, the perceived location of auditory stimuli can be markedly shifted when accompanied by a visual stimulus, due in part to the greater spatial resolution of the visual system (i.e., the “ventriloquist illusion” Pick et al., 1969; Welch and Warren, 1980; Bertelson and Radeau, 1981; Busse et al., 2005). In a similar vein, visual search times for targets that are redundantly coded in multiple dimensions tend to result in faster detection than those coded in a single dimension. Such “redundancy gain” effects in visual search are assumed to result from the integration of independent dimension-specific processing systems, such as orientation and color (e.g., Mordkoff and Yantis, 1993; Krummenacher et al., 2001, 2002; Feintuch and Cohen, 2002; Zehetleitner et al., 2009) and are thought to have an early pre-attentive perceptual locus (reviewed in Zehetleitner et al., 2008).

While the nervous system goes to great lengths to bind multisensory features presented in close spatial and temporal proximity (Ma and Pouget, 2008; reviewed in Stein and Stanford, 2008; Van der Burg et al., 2008), incompatibilities between information arriving at the different senses can have profound ramifications on both behavioral responses and perception. Tasks that pit incompatibilities between various stimulus inputs (e.g., Stroop, Flanker, and Simon tasks) have been extensively used in order to study numerous aspects of human information processing, including multisensory processing and perception (for review see De Gelder and Bertelson, 2003). Among the most robust observations resulting from this literature concerns the basic asymmetry that verbal and lexical information will interfere with sensory information (e.g., color), but not necessarily the converse (MacLeod, 1991). This relative dominance of word reading over other processes, such as color naming (Glaser and Glaser, 1982) or picture identification (Glaser and Dungelhoff, 1984), has led to accounts based on the degree of automaticity of certain abilities (LaBerge and Samuels, 1974; Shiffrin and Schneider, 1977; Cohen et al., 1992). According to such accounts, the more highly learned and automatic process of word reading interferes with more effortful and less practiced processes (e.g., color naming) that require greater attentional control.

In the present paper we fuse these two lines of reasoning (redundancy gains and stimulus-response conflict) in a crossmodal conflict task to investigate the relative strength and reliability of different visual features, when presented by themselves and when presented in additive combination. Specifically, we wished to determine if the perceptual estimates provided by visually presented words, visually presented colors, and their additive combination (color words in the same colored font) influenced the pattern behavioral conflict effects observed in a crossmodal Stroop task.

For this purpose we extended a variant of our previous crossmodal Stroop-SOA task (Donohue et al., 2013) to include three different combinations of visual cues. In our original variant of this task visual and auditory stimuli were separated by brief stimulus onset asynchronies (SOAs: −400 to + 400, in 100 ms increments) in order to study the time course of multisensory processing. Using this approach we observed that visual distractors produced larger incongruency effects on auditory targets than vice versa, and that these interacted with SOA to reveal larger effects when the irrelevant distractor occurred prior to the attended target, so-called “priming.” Further, we found that relative to neutral-stimuli, and across the wide range of SOAs employed, congruency led to substantially more behavioral facilitation than did incongruency to interference. By employing the SOA approach we have begun to map the time course of Stroop crossmodal interactions and thereby derived a unique platform by which to study the factors that influence sensory integration. In the current study we specifically tested how different combinations of visual features influence such integration processes.

**Figure 1:**
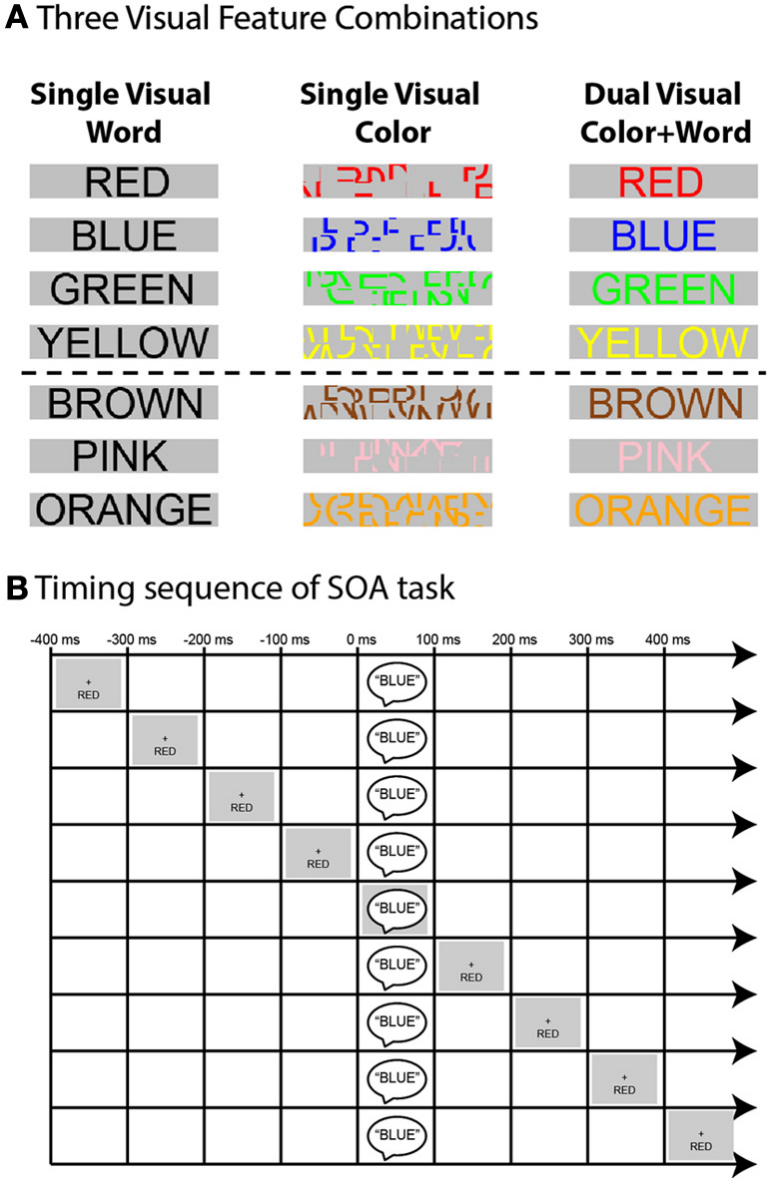
**(A)** Schematic depiction of the visual feature combinations used in the three tasks. The top four rows show visual stimulus component of the words that were used as targets (i.e., mapped to a response) while the bottom three rows show the neutral words (i.e., those not mapped to a response). **(B)** Schematic of SOA timing sequence. Example shown is that of an incongruent trial in the Single Visual Word task for the auditory attention condition wherein participants were instructed to report the auditory stimulus component (spoken-word “BLUE”) while ignoring the visual stimulus component (in this case the word “RED,” presented visually below fixation). The irrelevant visual information could come before or after the target in increments of 100 ms out to −400 and + 400 ms.

In the current experiment we tested three such visual feature combinations (Figure 1A). In addition to our original stimuli (Donohue et al., 2013) which were composed of auditory spoken words and visually presented color-words written in black font (*Visual Word Alone*), visual stimuli were presented in two other arrangements. In the first of these, the visual stimulus was composed of colored line segments deconstructed from scrambled color-words (*Visual Color Alone*), thereby showing a salient color stimulus that was devoid of sematic meaning. In the final condition, these two visual cues are combined and the visual stimuli were presented as fully formed color-words, presented in the corresponding or differently colored font (*Dual Visual Word + Color*). In the present paper we focused our interests on the main effects and interaction of the feature-combinations across the three tasks on behavioral performance, and refer the reader to (Donohue et al., 2013) for more information about the influences of the other experimental factors.

Based on the MLE framework described above we would expect that the additive combination of the *Dual Visual Word + Color* stimuli would evoke larger incongruency effects than either of the two individual visual features alone. In addition, based on the widely reported asymmetry between incongruency effects evoked by highly automatic word-naming and less practiced color-naming (reviewed in MacLeod, 1991), we would expect that when attending for auditory targets the visual words would evoke greater incongruency effects than would visual color stimuli.

## Methods

### Participants

Forty-eight healthy volunteers are included in the final analysis for this study (mean age = 21.5 years, 25 females, 6 left-handed). Four additional participants were excluded from the final analysis due to poor performance or failure to adhere to the task instructions. All participants were native English speakers with normal visual acuity and normal color vision. Participants were paid $15 per hour for their time. All methods were approved by the Institutional Review Board at Duke University.

### Stimuli

Experimental stimuli consisted of auditory spoken words and visually presented written words (Figure 1A). Auditory stimuli were the spoken words “Red,” “Blue,” “Green,” “Yellow,” “Pink,” “Brown,” and “Orange.” The words were recorded from a male native English speaker and were constrained to have an average duration of 385 ms. The auditory stimuli were presented centrally through two speakers positioned to the left and right of the CRT monitor and were played at a loudness of 50 dBSL.

Visual stimuli consisted of three different color-word and font-color combinations. The color-words were “RED,” “BLUE,” “GREEN,” “YELLOW,” “PINK,” “BROWN,” and “ORANGE” printed in Ariel font on a gray background. These stimuli appeared in the font colors red, blue, green, yellow, pink, brown, orange, and black depending on the experimental condition. The center of the words was 3.75° below fixation, with participants seated 57 cm from the CRT monitor. The visual stimuli were presented for 385 ms. Participants were instructed to maintain fixation on a central, white fixation-cross that remained on the screen for the duration of each ~3 min experimental run. A total of 10 runs, consisting of 108 trials each, were collected for each participant and participants were given the opportunity to rest between the runs.

### Experimental design and procedure

In the present experiment we were interested in determining if the perceptual estimates provided by visually presented words, visually presented colors, and their additive combination (color words in the same colored font) influenced the pattern of behavioral conflict effects observed in a crossmodal Stroop task. For this purpose we expanded upon our previous Stroop-SOA design (Donohue et al., 2013) to include three different visual feature combinations. In the following section we describe the 4 experimental factors that constitute the initial design, and end with the fifth factor, *Visual Feature Combination* that is the critical experimental manipulation in this study.

The current experimental design consisted of four independent variables that were varied for each subject and a fifth variable that was varied across subject groups. The first within-subject independent variable was “*Incongruency,”* which was defined by the correspondence between the color feature given by the auditory and visual stimuli on each trial. In all experimental sessions the stimuli consisted of three equally frequent configurations that comprised congruent, incongruent, and neutral trial types. In a congruent trial type, the color information provided by auditory and visual stimuli matched. The incongruent trial types consisted of auditory and visual stimuli that did not match, but for which there was a specifically assigned response mapping for the non-corresponding incongruent stimulus component. The neutral trials consisted of auditory and visual stimuli that did not match, but for which the irrelevant stimulus component was not mapped to one of the 4 response buttons.

The second within-subject independent variable, shown graphically in Figure 1B, was the “*Stimulus Onset Asynchrony”* or *“SOA”* between the presentation of the auditory and visual stimulus components. There were nine levels of SOA; −400, −300, −200, −100, 0, + 100, + 200, + 300, and + 400 ms, so that the task-irrelevant stimulus component could precede the target, occur simultaneously with it, or follow it. A total of 36 trials were presented within each SOA and incongruency condition, and the SOA and incongruency pairings were randomized across trials^1^. The inter-trial interval (i.e., the time between the first) stimulus component of two successive trials) was jittered randomly between 1600 and 1800 ms.

The third within-subject independent variable was the “*Attended Modality*.” During half the runs of each experimental session participants were instructed to attend to the auditory modality and report the identity of the auditory word with a button press while ignoring the visual stimuli. On the other half of the runs participants were instructed to attend to the visual modality, report the visual stimulus color with a button press, and to ignore the auditory stimuli. The order of the attended modality was randomized across runs and counterbalanced across participants. Across both attended modalities, the relevant-modality target stimulus features (i.e., those to which a response was mapped) were the words “Red,” “Green,” “Blue,” and “Yellow,” and the neutral words to which a response was not mapped were “Brown,” “Pink,” and “Orange.”

The fourth within-subject independent variable was the “*Response Button Mapping.”* To control for any confounds with specific target colors being mapped to specific buttons, we used two different response mappings in these experiments. For half of the participants the target words “Red,” “Green,” “Blue,” and “Yellow” were mapped to the “D,” “F,” “J,” and “K” keys, respectively. For the other half of the participants this mapping was flipped (left-to-right hand and index-to-middle finger) such that the mappings were to the “K,” “J,” “F,” and “D” key, respectively. Participants utilized both hands to respond with their index and middle fingers positioned on the keyboard as if they were typing. Planned analyses on the RT and error rates revealed that performance did not differ as a function of the assigned button mappings. All subsequent analyses were therefore, collapsed over this factor.

The fifth independent variable was the “*Visual Feature Combination*.” This variable was administered with three different levels to three different groups of 16 experimental participants. In the three different experimental sessions the visual stimuli were presented such that they contained semantic color information only, physical color information only, or both. In the *“Single Visual Word”* condition, the visual stimulus consisted of color-words written in a black front. Here the color information provided by the visual target stimulus was therefore expressed by the semantic meaning of the word only. In the *“Single Visual Color”* condition, visual stimuli consisted of scrambled versions of the color-words presented in font colors corresponding to the various color options. Because the scrambling process destroyed the semantic content of these stimuli, the visual target information in this condition was expressed solely by the physical font color of the scrambled line segments. In the “*Dual Visual Color + Word”* condition, the visual stimuli consisted of written color-words that were presented in the matching font color (e.g., the word GREEN written in a green font color). For these stimuli the visual target information consisted of both the semantic meaning of the words and the matching physical font color.

### Behavioral analysis

Behavioral responses were monitored and recorded while participants performed the task. Trials were counted as correct if the subject responded correctly between 200 and 1200 ms following the presentation of the target stimulus. As no systematic behavioral differences were observed for the four different target colors or for the order of button-response mappings (*p*'s > 0.05), data were collapsed over the different colors and response mappings to arrive at within-participant mean response times (RTs; correct trials only) for the other levels of the remaining factors. RTs were then submitted to a 4-way mixed-model analyses of variance (ANOVA), with the within-subject factors of *Incongruency* (3 levels; congruent, neutral, incongruent), *SOA* (9 levels), *Attended Modality* (2-levels; visual and auditory), and the between-subject factory of *Visual Feature Combination*. In order to ensure that any effects of incongruency we were observing were not due to the fact that one modality had overall slower RTs than the other (auditory being slower than visual), we conducted an additional analysis on the data following a modality-normalization procedure. For this purpose the RTs for each level of congruency and SOA were divided by the mean RT for all conditions in each modality. These normalized data were then entered into the same ANOVAs as described above.

Further, to examine the specific effect of visual feature combination, we looked at facilitation (neutral vs. congruent), interference (incongruent vs. neutral), and full congruency (incongruent vs. congruent) effects across the various SOAs for each of the Visual Feature Combinations. Additional two-tailed, paired *t*-tests were performed on specific planned comparisons and are described in more detail in the appropriate Results sections below. The significance thresholds were set to a *p*-value of 0.05 and, when applicable, adjusted using the Greenhouse-Geisser correction for non-sphericity. Partial eta-squared values (η^2^_*p*_) are reported as an additional metric of effect size for all significant or marginally significant (0.1 > p > 0.05) ANOVA contrasts. Accuracy was very high across all conditions and tasks (mean = 94.2%) and did not differ as a function of Attended Modality (*p* = 0.81) or the Visual Feature Combination (*p* = 0.26). We therefore, restricted our subsequent analyses to only the reaction time data.

## Results

**Figure 2:**
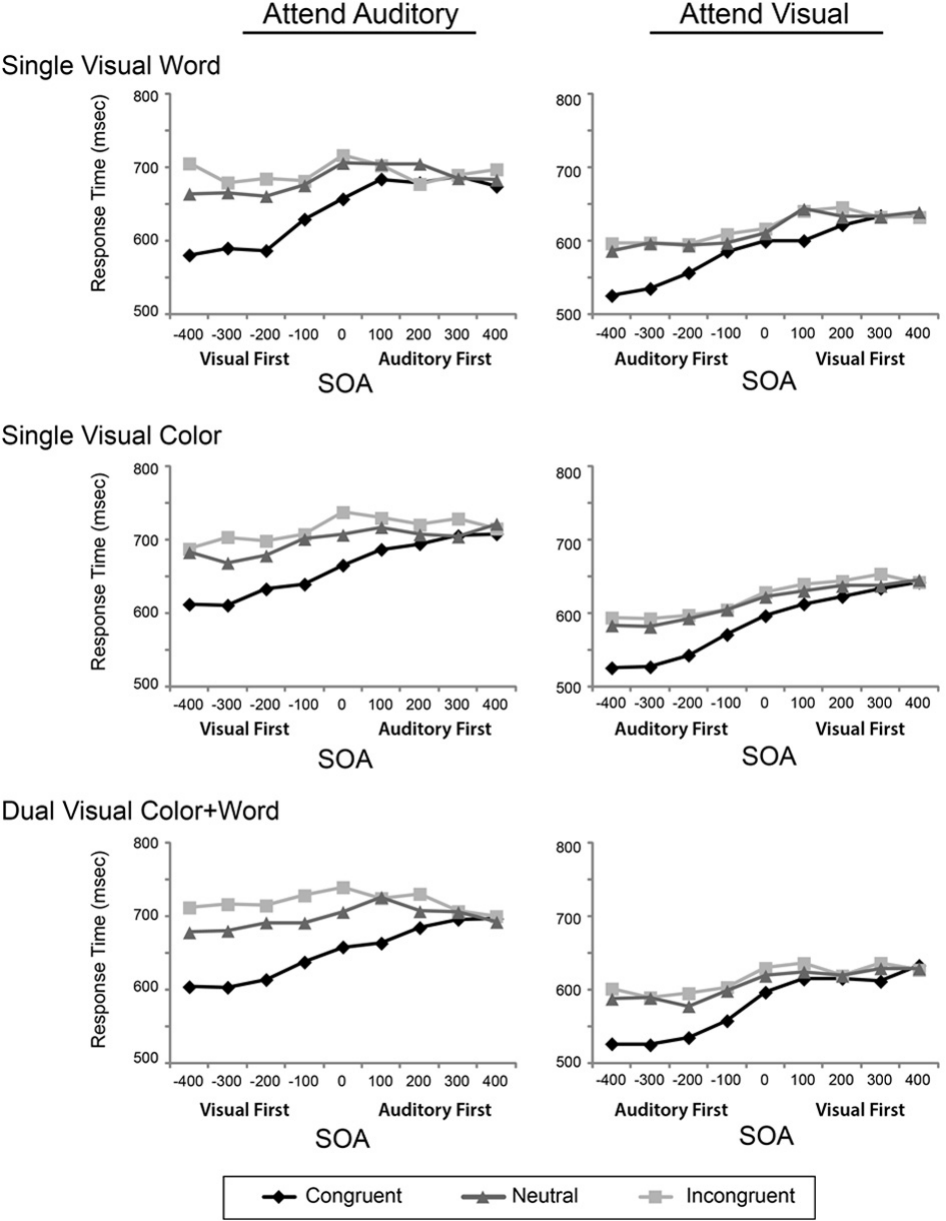
**Reaction times for the congruent, neutral, and incongruent conditions are plotted across the 9 SOAs, presented in separate rows for each visual feature combination, and in separate columns for the two attentional modalities**.

**Figure 3:**
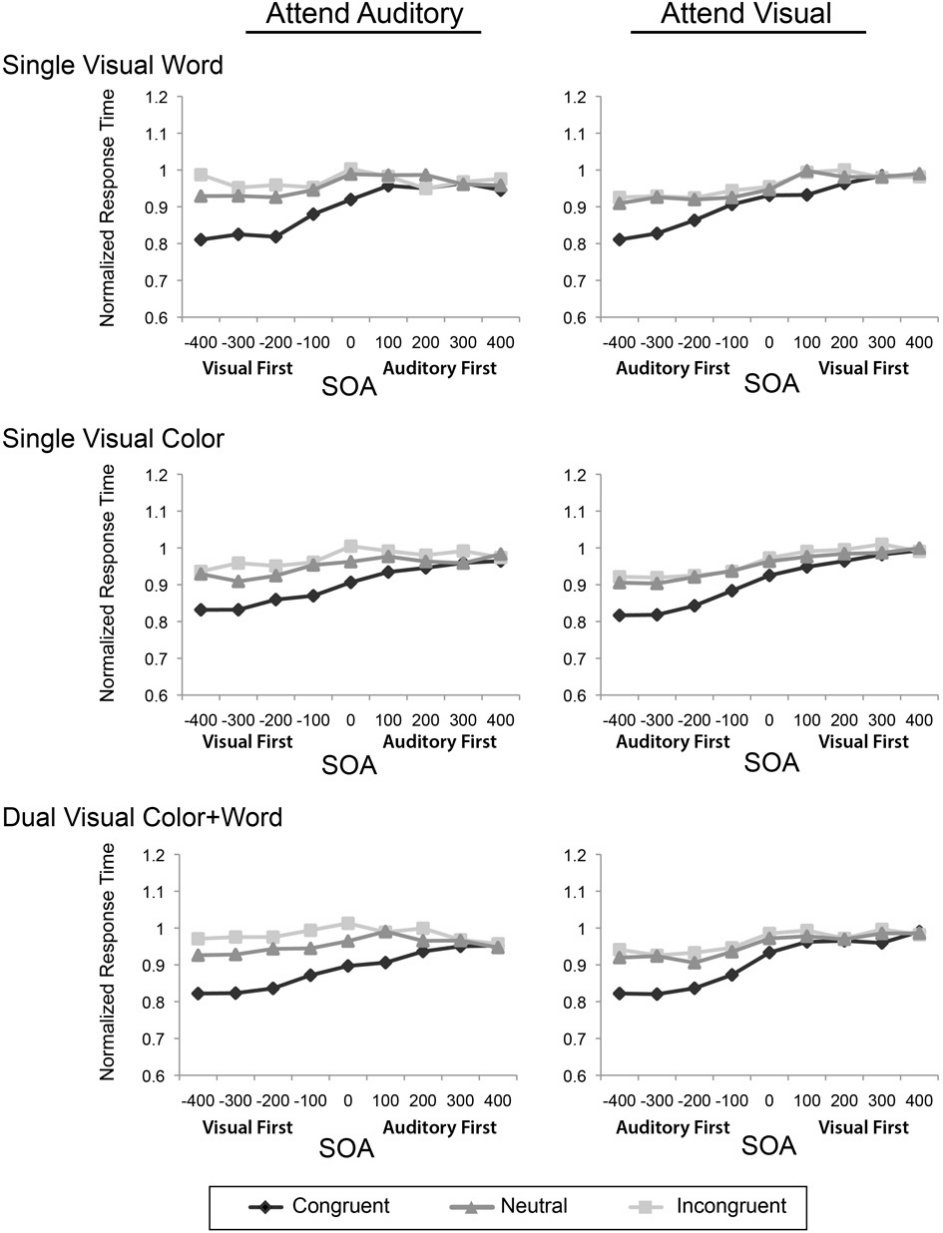
**Modality-normalized reaction times for the congruent, neutral, and incongruent conditions are plotted across the 9 SOAs, presented in separate rows for each visual feature combination, and in separate columns for the two attentional modalities**.

To assess the influence of the experimental design factors on multisensory Stroop conflict, we conducted a mixed-model ANOVA with the within-subject factors of Attended Modality, SOA, and Incongruency and the between-subject factor of Visual Feature Combination on the reaction times. Two analyses of variance were performed, one on the raw reaction times (Figure 2), and second test on the modality-normalized reaction times (Figure 3).

The ANOVA performed on the raw reaction times revealed a main effect of Modality [*F*_(1, 45)_ = 121.40, *p* < 0.001, η^2^_p_ = 0.73], a main effect of SOA [*F*_(2.99, 134.69)_ = 86.00, *p* < 0.001, η^2^_p_ = 0.66], and a main effect of Incongruency [*F*_(1.59, 71.73)_ = 273.44, *p* < 0.001, η^2^_p_ = 0.84]. This ANOVA also revealed significant interactions between SOA and Incongruency [*F*_(10.10, 454.53)_ = 24.61, *p* < 0.001, η^2^_p_ = 0.35], Attended Modality and SOA [*F*_(4.38, 197.23)_ = 5.08, *p* < 0.001, η^2^_p_ = 0.10], Attended Modality and Incongruency [*F*_(1.78, 80.17)_ = 17.33, *p* < 0.001, η^2^_p_ = 0.03], and a three-way interaction between Attended Modality, SOA, and Incongruency [*F*_(11.02, 496.01)_ = 2.10, *p* = 0.02, η^2^_p_ = 0.05]. Collectively these results indicate that visual stimuli are processed faster than auditory stimuli and replicate previous findings related to congruency and SOA acquired in a different pool of participants using only the single visual word condition (Donohue et al., 2013). These findings are discussed briefly below and in greater detail in the Donohue et al. article.

Although the general pattern of results showed that the irrelevant visual stimuli had more of an influence on the processing of auditory stimuli, the auditory stimuli were also processed more slowly than the visual stimuli. As such, any increased incongruency effects may be mainly resulting from these modality differences in processing speeds. To adjust for the contribution of these baseline differences, we normalized the data using the mean RTs for each modality and re-computed the same ANOVAs as above. This analysis revealed that there was still a main effect of SOA [*F*_(2.95, 45)_ = 92.57, *p* < 0.001, η^2^_p_ = 0.673], a main effect of incongruency [*F*_(1.51, 67.94)_ = 243.95, *p* < 0.001; η^2^_p_ = 0.844], an interaction of SOA and incongruency [*F*_(9.9, 445.73)_ = 26.12, *p* < 0.001; η^2^_p_ = 0.367], an interaction of SOA and modality [*F*_(4.56, 205.41)_ = 7.6, *p* < 0.001; η^2^_p_ = 0.144], and an interaction of attended modality and incongruency [*F*_(1.8, 80.94)_ = 11.49, *p* < 0.001; η^2^_p_ = 0.203]. There was no main effect of attended modality, confirming the normalization. Together, these data indicate that while attending to and discriminating the auditory stimulus components slowed RTs generally, the asymmetric pattern of facilitation and incongruency effects do not simply reflect differences in the baseline processing speeds for the two modalities.

Both the original and normalized results confirmed our previous findings (Donohue et al., 2013); however, the pattern of results from these omnibus ANOVAs failed to reveal any robust effects or interactions of our primary variable of interest, Visual Feature Combination. Although this factor did not produce a significant main effect, two-way interaction, or three-way interaction, it did result in a marginal four-way interaction of Attended Modality by SOA by Incongruency by Visual Feature Combination, that weakly trended toward significance in both the original [*F*_(16, 729)_ = 1.46, *p* = 0.08, η^2^_p_ = 0.06] and normalized [*F*_(22.36, 503.21)_ = 1.41, *p* = 0.1, η^2^_p_ = 0.06] data. In light of this trending interaction, we therefore, conducted more focused analyses on the attended auditory data, where the greatest feature combination differences appeared in order to determine if the redundancy of visual features in this condition produced behavioral effects that were obscured in the omnibus analysis that included all of the conditions.

First, considering all three levels of congruency, we found a marginal three-way interaction between SOA, Incongruency, and Visual Feature Combination in the original [*F*_(21.36, 480.63)_ = 1.45, *p* = 0.09, η^2^_p_ = 0.06] and normalized data [*F*_(20.94, 471.24)_ = 1.44, *p* = 0.09, η^2^_p_2 = 0.06], suggesting a possible influence of redundant visual features. Focusing on the Interference (neutral minus incongruent) and Facilitation (congruent minus neutral) effects separately, for just the attended auditory task, revealed a marginal two-way interaction for Interference [Oiriginal data: *F*_(13.45, 302.60)_ = 1.65, *p* = 0.07, η^2^_p_ = 0.07; Normalized Data: *F*_(13.57, 305.25)_ = 1.65, *p* = 0.07, η^2^_p_ = 0.07] with greater overall interference for the dual visual than either of the single visual conditions, but no main effects or interactions for Facilitation (all *p*'s > 0.45). Further ANOVA on the attended visual modality revealed no main effects of or interactions with Visual Feature Combination (all *p*'s > 0.6). Tests looking at only Facilitation and Interference, as above, also revealed no main effects, nor interactions with Visual Feature Combination (all *p*'s > 0.35).

To further ensure that we were not missing any effects with Visual Feature Combination due to the large amount of factors in our ANOVA, we repeated the analysis limiting ourselves first to the negative SOAs (where the biggest effects were in general). This revealed no significant effects or interactions of Visual Cue Combination with any of the other factors (Attended Modality, SOA, Congruency). Further, restricting our analysis to just the 0 SOA (as might occur in a traditional conflict task) did not reveal any significant main effects or interactions with Visual Cue Combination. Collectively, these analyses therefore, provide only little evidence that redundant visual features may lead to differential behavioral incongruency effects relative to singleton features.

## Discussion

Here we set out to test for stimulus redundancy effects in a crossmodal Stroop conflict task. Based on theoretical constructs such as the MLE framework (e.g., Beierholm et al., 2009) and redundancy gains in visual search (reviewed in Zehetleitner et al., 2008) that show consistent behavioral benefits of redundant sensory-features, we expected that redundant visual feature information would lead to altered incongruency effects as compared to single features presented alone. Such an alteration could in principle take two forms. In the case of auditory attended targets, one would expect the dual visual stimuli would lead to a greater behavioral influence, and thus, larger incongruency effects, than either of the individual visual features alone. When the task was to attend to the visual stimulus, however, one would expect that the improved visual sensory representation resulting from redundant visual features would allow for less influence by the irrelevant auditory distracters, thus, leading to reduced incongruency effects relative to the individual-visual-feature conditions. Unexpectedly, however, we did not see significance evidence for either of these patterns of effects.

Before discussing this surprising lack of redundancy effects in the current results, it is worth first mentioning that the present findings demonstrate a close replication of our previously reported results that had tested only the *Visual Word Alone* stimuli in a separate pool of participants (Donohue et al., 2013). As observed previously, visual distracters in the current experiment produced larger and longer lasting incongruency effects on auditory targets than vice versa. For both attentional modalities, stimulus incongruency interacted with SOA, yielding larger incongruency effects when the irrelevant distracter occurred prior to the attended target, and reduced but still significant effects when the irrelevant distracter followed the target (i.e., “priming” and “backward interference,” respectively, as also reported in Appelbaum et al., 2009, 2012; Ziai et al., 2011). In addition, under such multisensory stimulus conditions, congruent stimuli led to substantially more behavioral facilitation than incongruent stimuli led to interference, as reflected by comparison to the neutral stimuli. These same general patterns of priming and backward interference also held for the *Visual Color Alone* and the *Dual Visual Word + Color* conditions, as well as for both of the attended modalities.

The present study, however, was focused on the potential for differences between the three visual-feature combinations and how these may alter the pattern of behavioral incongruency effects. This contrast, however, yielded no main effect, nor any 2-way or any 3-way interactions. A marginal 4-way interaction was present that in *post-hoc* analyses appeared to be primarily driven by a weak trend for a difference in the amount of interference (neutral vs. incongruent) for the attended auditory task only. Collectively, these findings indicate very little difference in the pattern of behavioral effects produced across the three visual feature combinations.

As noted above, the relative lack of interaction is surprising because of other existing evidence for redundancy gains (e.g., crossmodal integration as reported by Bertelson and Radeau, 1981; and visual search task as reviewed in Zehetleitner et al., 2008). Under this logic the redundant visual information, in the form of a physical color and a semantic color-word, should lead to stronger processing of the visual representation of the target color at the expense of the auditory representation. This would have been expected to create an asymmetry in the incongruency effects for the two attentional conditions such that redundant visual features would have biased responses to favor the visual features at the expense of the competing auditory response. Similarly, based on widely reported asymmetries between interference induced by the more highly-learned and automatic process of word reading, vs. the more effortful and controlled process of color-naming (Glaser and Glaser, 1982; Durgin, 2000; Appelbaum et al., under review), one would also expect greater incongruency effects in the *Visual Color Alone* than the *Visual Word Alone* conditions. Nonetheless, as indicated by the relative lack of such effects, no such lexical/perceptual asymmetry was at play in these cross-modal incongruency interactions. While it is important to consider that the use of lexically meaningful neutral stimuli may have altered the relative ratio of facilitation-to-interference (Brown, 2011), it was still observed that these ratios did not differ over the three Visual Cue Combination conditions. These findings therefore, suggest that, unlike crossmodal integration and visual search, crossmodal conflict of the kind employed in this study is less influenced by stimulus redundancy.

One potential explanation for this symmetry may lie in the particular stimuli themselves. In the preponderance of cases where MLE applies, near-threshold stimuli are used. Such stimuli result in substantial uncertainty about the identity of one or more modalities, and therefore, redundancy may serve a particularly useful role in “anchoring” perception under those circumstances. For example, the spatial resolution of auditory stimuli is much less than that of visual stimuli, and thus, in cases such as the “ventriloquist illusion” (Pick et al., 1969; Welch and Warren, 1980; Bertelson and Radeau, 1981) ambiguity about the location of the auditory signal engenders a shift in the auditory perceptual localization toward the more spatially reliable visual stimulus (e.g., “auditory driving” Gebhard and Mowbray, 1959; Shipley, 1964). In contrast, in the present tasks, the visual stimuli were all supra-threshold and easy to perceive, as indicated by both relatively fast RTs and high response accuracy. Given this, while the redundancy of visual features in the *Dual Visual Word + Color* condition certainly added to the perceptual signal, it was done to an already robust stimulus and therefore, was more likely to be tautological. Such highly salient stimuli, therefore, may not have left any room for the added redundancy to improve performance. Future research using degraded visual stimuli, or experiments in which the exposure to previously unlearned stimuli are explicitly manipulated to alter the expected reliability (as in typical MLE designs), may be able to determine if saliency played a particularly important role here (see Yuval-Greenberg and Deouell, 2009 for relevant examples).

In conclusion, while we find that multisensory conflict is modulated by the attended modality of the target, and by the SOA between targets and distracters, we find only minimal evidence for redundancy gains. We interpret these findings to indicate that in the context of multisensory conflict, semantic (word) features, perceptual (color) features, and the combination of both provide sufficiently reliable estimates of the visual stimulation as to evoke equivalent behavioral incongruency effects.

### Conflict of interest statement

The authors declare that the research was conducted in the absence of any commercial or financial relationships that could be construed as a potential conflict of interest.

## Footnotes

^1^In our previous study (Donohue et al., 2013) we did not observe any difference in behavioral effects for randomized and blocked SOA arrangements and therefore used only the randomized SOA arrangement in the present study.
